# Parameters associated with design effect of child anthropometry indicators in small-scale field surveys

**DOI:** 10.1186/s12982-016-0054-y

**Published:** 2016-12-07

**Authors:** Erin N. Hulland, Curtis J. Blanton, Eva Z. Leidman, Oleg O. Bilukha

**Affiliations:** Emergency Response and Recovery Branch, Division of Global Health Protection, Centers for Disease Control and Prevention, Atlanta, GA 30333 USA

**Keywords:** Design effect, Anthropometry, Survey methodology, Cluster surveys, Wasting, Stunting, Underweight

## Abstract

**Background:**

Cluster surveys provide rapid but representative estimates of key nutrition indicators in humanitarian crises. For these surveys, an accurate estimate of the design effect is critical to calculate a sample size that achieves adequate precision with the minimum number of sampling units. This paper describes the variability in design effect for three key nutrition indicators measured in small-scale surveys and models the association of design effect with parameters hypothesized to explain this variability.

**Methods:**

380 small-scale surveys from 28 countries conducted between 2006 and 2013 were analyzed. We calculated prevalence and design effect of wasting, underweight, and stunting for each survey as well as standard deviations of the underlying continuous Z-score distribution. Mean cluster size, survey location and year were recorded. To describe design effects, median and interquartile ranges were examined. Generalized linear regression models were run to identify potential predictors of design effect.

**Results:**

Median design effect was under 2.00 for all three indicators; for wasting, the median was 1.35, the lowest among the indicators. Multivariable linear regression models suggest significant, positive associations of design effect and mean cluster size for all three indicators, and with prevalence of wasting and underweight, but not stunting. Standard deviation was positively associated with design effect for wasting but negatively associated for stunting. Survey region was significant in all three models.

**Conclusions:**

This study supports the current field survey guidance recommending the use of 1.5 as a benchmark for design effect of wasting, but suggests this value may not be large enough for surveys with a primary objective of measuring stunting or underweight. The strong relationship between design effect and region in the models underscores the continued need to consider country- and locality-specific estimates when designing surveys. These models also provide empirical evidence of a positive relationship between design effect and both mean cluster size and prevalence, and introduces standard deviation of the underlying continuous variable (Z-scores) as a previously unexplored factor significantly associated with design effect. The magnitude and directionality of this association differed by indicator, underscoring the need for further investigation into the relationship between standard deviation and design effect.

## Background

In humanitarian emergencies, information on nutritional status of the affected population, particularly children aged 6–59 months, is frequently used to determine the severity of the situation and to monitor progress of key life-saving interventions. Cross-sectional surveys are commonly used in these settings to obtain representative estimates of wasting [1]. While the accepted gold standard of cross-sectional surveys is the simple or systematic random sampling method (SRS), in humanitarian emergencies, where up-to-date lists may not exist and populations are dispersed, SRS is often too costly or logistically unfeasible [2]. Therefore, in humanitarian settings, small scale cluster surveys are more commonly undertaken. These surveys are designed with the emergency context and rapid need for information in mind. Likewise, geographic scope is small, usually a group of refugee camps, or an affected district or livelihood zone, which allows for a simple two-stage design. Samples are designed to be approximately self-weighted to simplify analysis, and sample size is usually within a range of 300–900 children aged 6–59 months in order to reduce cost and time in the field.

Cluster sampling has been accepted as a valid alternative to SRS in these and other settings, and is also routinely used in large-scale demographic surveys including UNICEF’s Multiple Indicator Cluster Survey (MICS) and USAID’s Demographic and Health Survey (DHS) [3, 4]. To account for the loss of precision resulting from increased within-cluster homogeneity in the sample due to the complex sampling design, researchers adjust the required sample size using a design effect, a ratio of the variance under the complex design to the variance under SRS assuming equal cluster size [2, 5, 6].

Design effect (DEFF) is a function of the mean cluster size in the survey and the intracluster correlation coefficient (ρ), a measure of the between-cluster variance as a proportion of the total variance, and acts as a direct multiplier of sample size in order to achieve the same precision as under SRS. The most widely used equation for calculating DEFF is as follows [7]:

$$DEFF = 1 + \rho *\left( {B - 1} \right)$$

where *ρ*—the intracluster correlation coefficient, and B—the mean cluster size.

Previous research has demonstrated that DEFF varies from one health outcome to the next as the expected clustering increases: DEFFs of 1.0–2.0 are common for most nutrition indicators while programmatic indicators, such as measles coverage or access to safe water sources, can have DEFFs greater than 10.0 [5, 8]. For nutrition surveys, a default DEFF of 2.0 was first recommended by the United Nations Administrative Committee on Coordination/Sub-Committee on Nutrition (ACC/SCN) in 1994 in accordance with the ‘30 × 30’ design for cluster surveys, which were designed to reliably provide estimates of wasting, stunting, and underweight with a precision of ±5% [9, 10]. This design called for using a pre-determined sampling design of 30 clusters with 30 children each, resulting in a set sample size of 900 children [9]. After years of implementation, it was observed that the DEFF of 2.00 used in the planning of these surveys was often overestimated when compared to what was calculated after implementation. As illustrated in the following equation, an estimate of the expected DEFF is used in determining sample size needed for a small-scale cluster survey [7]:

$$n = \frac{{p\left( {1 - p} \right)t^{2} }}{{d^{2} }}*DEFF$$

where p—the estimated prevalence of the outcome of interest (usually wasting); t—a Student’s t-score with degrees of freedom equal to the number of clusters minus 1 and an alpha of 0.05 (corresponding to 95% confidence level); d—half-width of the two-sided 95% confidence interval; DEFF—design effect, and n—target sample size.

As DEFF is a direct multiplier of sample size in the above equation, an overestimate of DEFF results in a larger sample size than required for a given precision, and consequently increased cost and duration of the survey [9]. In 2006, Standardized Monitoring and Assessment of Relief and Transitions (SMART) guidelines were released with a recommendation to calculate sample size using an estimated DEFF and other predictors specific to the study setting, a contrast to the preceding guidance prescribing a sample size of 900 children [11]. These new guidelines thereby necessitated an improved understanding of observed DEFF in different settings. The emphasis by the SMART initiative on calculating sample size has resulted in more consistent reporting of observed DEFF since its introduction in 2006 [5]. The first aim of this study was therefore to review available anthropometric surveys to describe the magnitude and variability of DEFFs to help guide survey planning.

The second aim of this study was to evaluate factors associated with DEFF. A positive relationship between mean cluster size and DEFF is derived from the mathematical formulae, although there is little empirical evidence confirming this relationship [12]. Prevalence has also been shown to be associated with DEFF, with a maximum value of DEFF at 50% prevalence [2]. Prevalence is a parameter in equations for both sample size and DEFF (via the intracluster correlation coefficient) [7]. We further hypothesized that other parameters may also be associated with DEFF, including the standard deviation (SD) of Z-scores. Z-scores are a measure of the nutritional status of a child, expressed as the number of SDs below or above a reference median value [13, 14]. Age- and sex-specific reference values are most commonly obtained from the 2006 WHO growth standards [15]. Previous research has demonstrated that Z-scores within a population are normally distributed with a SD of approximately 1.0; the shape of the distribution does not vary based on the nutritional status of the population, as measured by the mean Z-score [14]. Based on the finding that SD remains in a relatively narrow range for each indicator regardless of mean Z-score, WHO guidance recommends that the SD of Z-scores can be used as a data quality indicator as well as a measure of variability [14]. The introduction of random non-directional errors, such as those introduced when age is estimated rather than calculated or when teams are imprecise in measuring height or weight, can result in wider SD relative to the acceptable ranges outlined by WHO [13]. Conversely, Z-score distributions that are much narrower than the usually seen ranges suggest the possibility of falsified data. We therefore included SD of the Z-scores to assess the degree to which data quality in addition to variability impact DEFF in anthropometric surveys.

## Methods

Data for these analyses were obtained from Action Contre la Faim (ACF) International, an international humanitarian non-governmental organization that conducts multiple small-scale field nutrition surveys in humanitarian settings worldwide [16]. These data represent 394 surveys conducted between 2006 and 2013 [17]. Surveys with fewer than 25 clusters or sample sizes smaller than 196 persons were excluded a priori from all analyses as they did not meet minimum standards for small scale cluster surveys [18, 19]. Surveys larger than 1500 persons were excluded from all analyses as they are not considered small-scale.

All included surveys collected a minimum set of standard anthropometric indicators for each child including the sex, age (in months), height (in cm), and weight (in kg). Z-scores were calculated for each child for the three main nutrition indicators—Weight-for-Height (WHZ), Height-for-Age (HAZ), and Weight-for-Age (WAZ)—using the WHO 2006 growth standards [15]. For each of the three nutritional indices, the mean and SD were computed for each survey to describe the Z-score distribution. Prevalence of wasting, stunting, and underweight were derived from the continuous Z-score distributions for each survey wherein each reflects the proportion of children with Z-scores less than −2 for WHZ, HAZ, and WAZ, respectively. Separately for each indicator, outlier observations were excluded from a survey if the observed Z-score of a child fell outside the flexible exclusion range of ±4 Z-scores from the observed survey sample mean, as described by WHO [13]. Individual observations within each survey were also excluded for children without information on height, weight, age or sex [13]. To describe the survey design, we computed the mean, variance, median and interquartile range for the cluster size and number of clusters. Survey location and year were also recorded. Survey location was categorized into eight geographical groupings as seen in Table 1. While most of the groupings were done by region and encompassed multiple countries, Sudan and Democratic Republic of Congo were kept as their own categories due to a large number of surveys conducted in these two countries. All data were aggregated and cleaned using SAS Version 9.3 [20].

The DEFF was calculated for prevalence of wasting, stunting and underweight and using the same outlier exclusions. DEFFs lower than 1.0 were changed to 1.0 as the DEFF for a cluster survey is always higher than for SRS where DEFF is 1.0 [21]. To assess variability in the estimates, measures of central tendency and dispersion were calculated for DEFF by indicator. The percent of surveys with a DEFF below 2.0 and 1.5 were also computed. To assess changes in survey design and implementation during the study period, one-way ANOVA was used to quantify annual changes in the mean cluster size, number of clusters, and total sample size.

One main goal of our analysis was to model DEFF. Univariable models were run to observe the unadjusted relationship between DEFF and each predictor variable. For each of the multivariable models, we included the five predictors: prevalence, SD of the Z-scores, mean cluster size, survey location, and survey year. Survey year was modeled as a categorical variable as there was not a clear linear relationship between DEFF and survey year. Prevalence, SDs and mean cluster size were modeled as continuous linear terms; models with prevalence and SD as quadratic terms were considered but did not significantly improve model fit, thus the linear predictors were used for ease of interpretation. Generalized linear models with all five predictors of DEFF were run using SAS version 9.3 [20]. Model diagnostics including plotting full and Jackknife residuals, checking for points with high leverage and outliers, and assessing Cook’s distance for each point, were run in RStudio for each of the three models. Observations with significantly high leverage or Cook’s distance were removed from the multivariable analyses [22–27]. Surveys with a Z-score SD less than 0.8 were also excluded, separately for each model, to remove the possibility of including falsified data [13, 19, 28]. All figures were produced in RStudio [22]. Coefficients for prevalence and Z-score SDs were scaled to 0.1 unit increases for ease of interpretation.

## Results

A total of 394 surveys conducted between 2006 and 2013 in 28 different countries were examined for this study, as seen in Table 1. Fourteen surveys were excluded from the analysis: seven surveys had sample sizes greater than 1500 children, two surveys had sample sizes smaller than 196 children, four surveys had fewer than 25 clusters, and one survey had both fewer than 25 clusters and a sample size smaller than 196 children, yielding 380 surveys included for analysis. The median number of children per survey was 887.00 [Interquartile Range (IQR): 687.50–947.00].

**Table 1:** Number of surveys by location, country and exclusion criteria

Location	Country	Initial number of surveys	Number excluded due to SS <196	Number excluded due to <25 clusters	Number excluded due to SS >1500	Final number of surveys
Americas	Bolivia	1	0	0	0	1
	Guatemala	1	0	0	0	1
	Haiti	13	0	0	0	13
Central/Southern Africa	Angola	1	0	0	0	1
	Central African Republic	9	0	0	0	9
	Madagascar	1	0	0	0	1
	Chad	17	0	0	0	17
Democratic Republic of Congo^a^		129	0	3	0	126
East Africa	Burundi^b^	4	2	1	0	2
	Ethiopia	6	0	0	0	6
	Kenya	26	0	0	0	26
	Somalia	4	0	0	0	4
	South Sudan	6	0	0	0	6
	Uganda	18	0	0	6	12
Middle East	Pakistan	12	0	0	1	11
	Afghanistan	9	0	0	0	9
South Asia	Bangladesh	16	0	0	0	16
	India	1	0	0	0	1
	Myanmar	7	0	0	0	7
	Nepal	8	1	0	0	7
	Philippines	4	0	0	0	4
Sudan^a^		69	0	1	0	68
West Africa	Burkina Faso	5	0	0	0	5
	Guinea	5	0	0	0	5
	Mali	8	0	0	0	8
	Mauritania	2	0	0	0	2
	Niger	11	0	0	0	11
	Sierra Leone	1	0	0	0	1
Total		394	3^b^	5^b^	7	380

^a^Democratic Republic of Congo and Sudan had a much larger number of surveys than any other countries and were kept separate from larger regional groupings

^b^One survey had both fewer than 25 clusters and fewer than 150 children and was counted twice in the exclusion columns but only once in the initial and final numbers of surveys columns

### Predictor variables

The number of surveys varied by year with a maximum of 92 surveys conducted in 2008 and a minimum of 10 surveys conducted in 2013, as seen in Table 2. The number of surveys also varied by location, with both Sudan and Democratic Republic of Congo having more surveys than any other region, justifying the segregation of those two countries from larger regional groupings.

**Table 2:** Distribution of number of surveys by location and year

Survey location	Survey year								
	2006	2007	2008	2009	2010	2011	2012	2013	Total
Americas	0	0	5	8	1	1	0	0	15
Middle East	4	2	2	0	2	6	4	0	20
South Asia	3	2	4	2	6	7	3	8	35
Democratic Republic of Congo	18	13	32	23	20	14	6	0	126
Sudan	26	23	18	1	0	0	0	0	68
Central/Southern Africa	1	3	8	6	5	1	4	0	28
East Africa	11	8	13	7	4	7	6	0	56
West Africa	4	9	10	2	1	2	2	2	32
Total	67	60	92	49	39	38	25	10	380

Table 3 presents measures of central tendency and dispersion for the prevalence of wasting, stunting, and underweight as well as the SDs of the continuous Z-score distributions for weight-for-height, weight-for-age, and height-for-age across all surveys. Median prevalence of wasting (10%) was generally lower than that of underweight (27%) or stunting (42%). Furthermore, the highest reported prevalence for wasting was 38% while both underweight and stunting had maximum prevalences at or greater than 70%, as seen in Table 3. The median SDs of WHZ and WAZ were 1.03 (IQR: 0.99–1.08) and 1.04 (IQR: 0.97–1.11), respectively, lower than that of HAZ [1.23 (IQR: 1.14–1.31)].

**Table 3:** Distribution of anthropometric predictor variables (n = 380)

Predictor variable	Mean	SD	Median	IQR	Minimum	Maximum
*Wasting*						
Prevalence	0.12	0.07	0.10	0.06–0.17	0.00	0.38
SD of WHZ	1.04	0.08	1.03	0.99–1.08	0.84	1.33
*Underweight*						
Prevalence	0.28	0.12	0.27	0.19–0.36	0.05	0.70
SD of WAZ	1.04	0.10	1.04	0.97–1.11	0.78	1.37
*Stunting*						
Prevalence	0.40	0.18	0.42	0.29–0.54	0.03	0.79
SD of HAZ	1.22	0.15	1.23	1.14–1.31	0.56	1.68

**Fig. 1:**
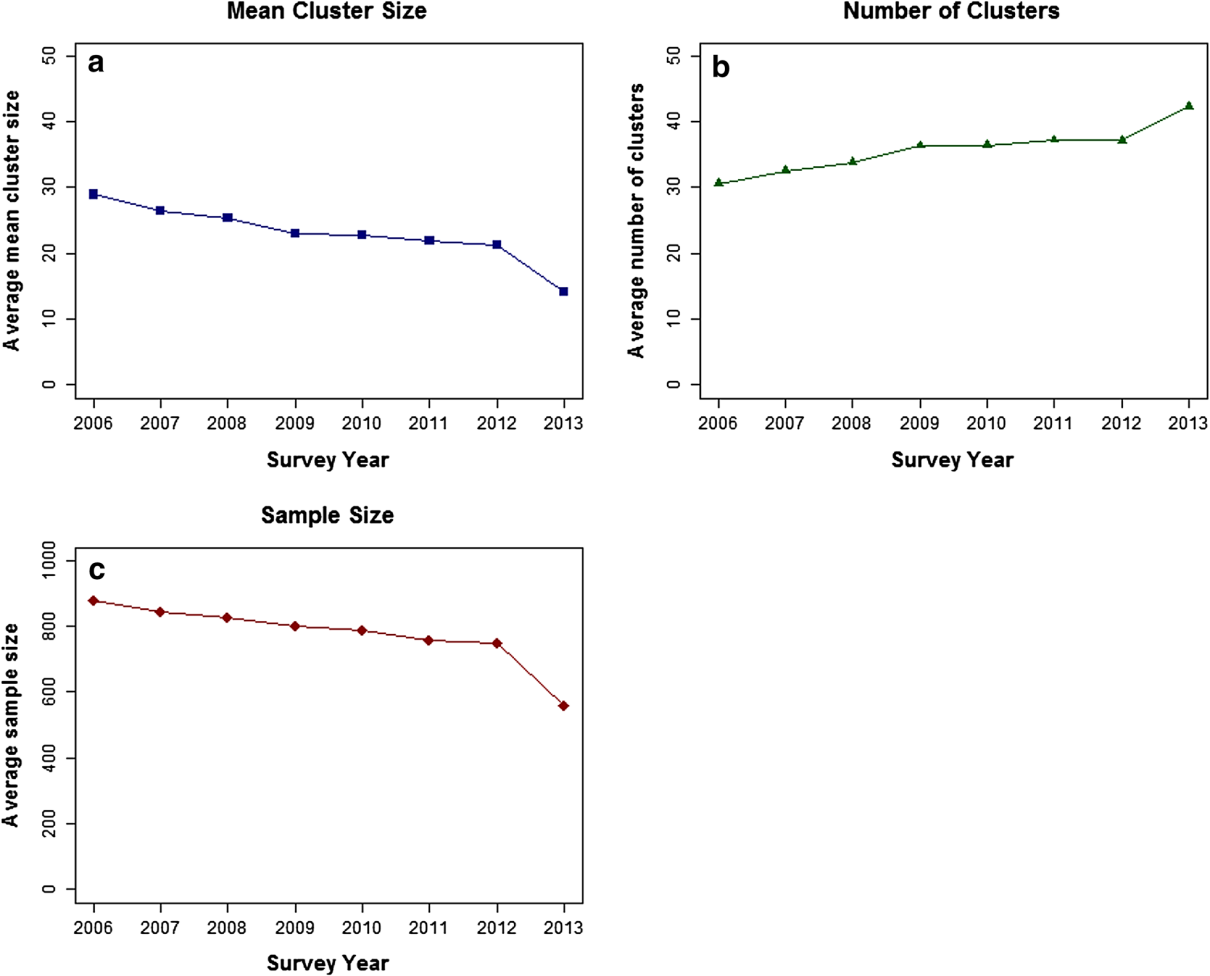
Trends in average mean cluster size (**a**), average number of clusters (**b**), and average sample size (**c**), 2006–2013

The surveys included had a smaller mean cluster size and larger mean number of clusters than prescribed by the formerly used ‘30 × 30’ design. The average mean cluster size between 2006 and 2013 was 24.68 children (median 26.90, range 6.90–59.88 children). The average number of clusters per survey was 34.40 (median 30.00, range 25.00–63.00 clusters). Both average cluster size and average number of clusters changed significantly over time (p < 0.001 for both). The average cluster size decreased from 28.93 (SD 5.50) in 2006 to 14.07 (SD 6.92) in 2013. The average number of clusters increased from 30.58 (SD 3.01) in 2006 to 42.30 (SD 13.20) in 2013. Over the same period, total sample size declined significantly from a mean of 878.24 children (SD 150.88) in 2006 to a mean of 556.50 children (SD 235.70) in 2013 (p < 0.001). These trends in the survey design during 2006–2013 are illustrated in Fig. 1.

### Design effects

**Fig. 2:**
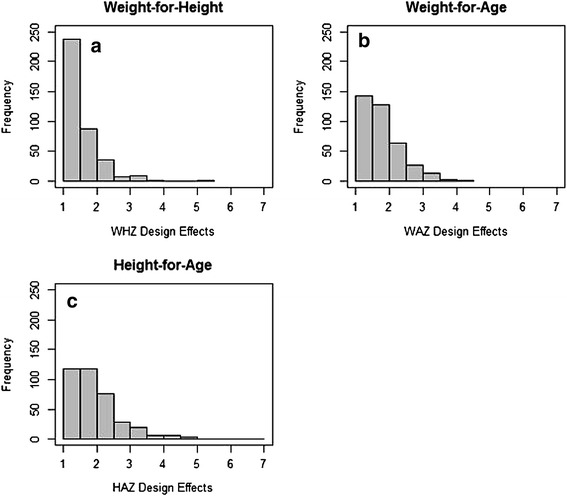
Distributions of design effects for wasting (**a**), underweight (**b**), and stunting (**c**)

The mean design effect for all three indicators fell below 2.00 (Table 4). Median DEFF for each of these three indicators was lower than the mean value, indicating a distribution skewed to the right. These right-skewed distributions are shown in the histogram plots in Fig. 2. The median DEFF for wasting (1.35) was lower than that for underweight (1.69), which was in turn lower than that for stunting (1.77). More than half of the DEFFs for underweight and stunting fell below 2.00, while this value exceeded 85% for wasting. Furthermore, the majority (63%) of DEFFs for wasting fell below 1.50.

**Table 4:** Distribution of DEFFs by indicator

Indicator	Mean	SD	Median	IQR	Minimum	Maximum	% below 2.00	% below 1.50
*Wasting*	1.50	0.54	1.35	1.10–1.72	1.00	5.21	85.79	62.63
*Underweight*	1.79	0.60	1.69	1.35 –2.08	1.00	4.46	71.32	37.63
*Stunting*	1.96	0.81	1.77	1.41–2.30	1.00	6.60	62.11	31.05

Median DEFF for wasting, stunting and underweight varied by region (Table 5). For all three indicators, DEFF was highest for surveys in the Middle East. For each region and year, the median DEFF for wasting was lower than that of underweight or stunting. Median DEFF for underweight was lower than that of stunting except in East Africa, the Americas, and for survey year 2010, where the two DEFFs were almost the same.

**Table 5:** Distribution of DEFFs by location and year

		Wasting	Underweight	Stunting
		Median (IQR)	Median (IQR)	Median (IQR)
*Location*	West Africa	1.28 (1.07–1.63)	1.51 (1.21–1.88)	1.57 (1.35–2.00)
	East Africa	1.38 (1.22–1.67)	1.63 (1.36–2.01)	1.62 (1.37–2.11)
	Central/Southern Africa	1.29 (1.07–1.49)	1.56 (1.38–1.85)	1.57 (1.28–2.12)
	Democratic Republic of Congo	1.36 (1.15–1.72)	1.77 (1.44–2.20)	1.78 (1.48–2.34)
	Sudan	1.46 (1.13–1.84)	1.60 (1.33–2.08)	1.87 (1.44–2.50)
	Middle East	1.71 (1.34–2.03)	1.99 (1.52–2.71)	2.31 (1.79–2.81)
	South Asia	1.17 (1.00–1.59)	1.44 (1.16–1.85)	1.61 (1.21–1.98)
	Americas	1.09 (1.00–1.58)	1.80 (1.28–2.17)	1.78 (1.50–2.47)
*Survey year*	2006	1.43 (1.10–2.05)	1.83 (1.40–2.29)	2.01 (1.48–2.77)
	2007	1.41 (1.25–1.75)	1.52 (1.29–1.97)	1.61 (1.38–2.20)
	2008	1.43 (1.17–1.71)	1.77 (1.44–2.25)	1.86 (1.51–2.42)
	2009	1.31 (1.06–1.55)	1.64 (1.28–2.00)	1.65 (1.36–2.07)
	2010	1.31 (1.11–1.58)	1.73 (1.36–1.99)	1.71 (1.29–2.09)
	2011	1.21 (1.00–1.47)	1.53 (1.32–1.87)	1.57 (1.23–1.93)
	2012	1.35 (1.17–1.70)	1.71 (1.41–2.20)	1.76 (1.43–2.81)
	2013	1.09 (1.00–1.15)	1.53 (1.14–1.82)	1.56 (1.15–1.91)

### Modeling

Results for the univariable and multivariable models for all three anthropometry indicators are presented in Table 6. For all multivariable models, outliers and observations with high leverage were excluded which resulted in exclusion of 2 observations from the wasting model, 1 observation from the underweight model and 4 observations from the stunting model. Additional observations, 2 for the underweight model and 5 for the stunting model, were excluded as they had an observed Z-score SD less than 0.8. The final models contained 378 observations for wasting, 377 for underweight, and 371 for stunting. Variance inflation factors (VIFs) were calculated for each model; no VIFs exceeded the standard cutoff of 10, and most met the criteria for low multicollinearity, with VIFs in the range of 1–5 [29, 30].

**Table 6:** Univariable and multivariable models of anthropometric DEFFs

Covariables		Univariable				Multivariable			
		Estimate	95% CI	p value	Type III p value	Estimate	95% CI	p value	Type III p value
*Wasting*									
Prevalence^a^		0.18	0.11 to 0.26	*<0.001*	*<0.001*	0.27	0.19 to 0.35	<*0.001*	<*0.001*
WHZ SD^a^		0.20	0.13 to 0.27	<*0.001*	<*0.001*	0.10	0.03 to 0.17	*0.009*	*0.009*
Mean cluster size		0.02	0.01 to 0.02	<*0.001*	<*0.001*	0.02	0.00 to 0.03	*0.013*	*0.013*
Location	West Africa	–	–	–	*0.003*	–	–	–	<*0.001*
	East Africa	0.21	−0.02 to 0.44	0.079		0.20	0.00 to 0.40	0.054	
	Central/Southern Africa	−0.02	−0.28 to 0.25	0.911		0.04	−0.19 to 0.28	0.710	
	Democratic Republic of Congo	0.19	−0.02 to 0.40	0.071		0.16	−0.05 to 0.38	0.141	
	Sudan	0.17	−0.05 to 0.40	0.127		−0.18	−0.40 to 0.04	0.104	
	Middle East	0.52	0.22 to 0.81	<*0.001*		0.40	0.14 to 0.67	*0.003*	
	South Asia	0.02	−0.23 to 0.28	0.868		0.27	0.03 to 0.51	*0.028*	
	Americas	−0.10	−0.43 to 0.22	0.532		0.29	−0.02 to 0.59	0.063	
Survey year	2006	–	–	–	*0.003*	–	–	–	0.102
	2007	−0.16	−0.34 to 0.03	0.092		−0.03	−0.19 to 0.13	0.723	
	2008	−0.17	−0.34 to −0.01	*0.040*		−0.05	−0.20 to 0.10	0.520	
	2009	−0.30	−0.49 to −0.10	*0.003*		−0.16	−0.35 to 0.03	0.109	
	2010	−0.31	−0.52 to −0.10	*0.004*		−0.23	−0.43 to −0.03	*0.028*	
	2011	−0.38	−0.59 to −0.16	<*0.001*		−0.29	−0.49 to −0.08	*0.006*	
	2012	−0.21	−0.45 to 0.03	0.094		−0.11	−0.34 to 0.11	0.313	
	2013	−0.56	−0.91 to −0.20	*0.002*		−0.29	−0.63 to 0.05	0.095	
*Underweight*									
Prevalence^a^		0.09	0.03 to 0.13	*0.001*	*0.001*	0.12	0.06 to 0.18	<*0.001*	<*0.001*
WAZ SD^a^		0.10	0.03 to 0.16	*0.002*	*0.002*	0.01	−0.07 to 0.08	0.834	0.834
Mean cluster size		0.02	0.01 to 0.03	<*0.001*	<*0.001*	0.03	0.02 to 0.04	<*0.001*	<*0.001*
Location	West Africa	–	–	–	*0.010*	–	–	–	*0.004*
	East Africa	0.08	−0.18 to 0.33	0.551		0.22	−0.03 to 0.48	0.091	
	Central/Southern Africa	0.05	−0.25 to 0.34	0.765		0.10	−0.18 to 0.39	0.479	
	Democratic Republic of Congo	0.27	0.04 to 0.50	*0.021*		0.03	−0.24 to 0.29	0.840	
	Sudan	0.12	−0.13 to 0.37	0.336		−0.04	−0.31 to 0.23	0.756	
	Middle East	0.51	0.18 to 0.84	*0.003*		0.52	0.20 to 0.84	*0.002*	
	South Asia	0.00	−0.28 to 0.28	0.990		0.10	−0.21 to 0.41	0.521	
	Americas	0.14	−0.23 to 0.50	0.462		0.50	0.12 to 0.87	*0.010*	
Survey year	2006	–	–	–	*0.010*	–	–	–	0.086
	2007	−0.26	−0.47 to −0.06	*0.013*		−0.20	−0.40 to −0.01	*0.045*	
	2008	−0.01	−0.19 to 0.18	0.928		0.05	−0.13 to 0.23	0.605	
	2009	−0.24	−0.45 to −0.02	*0.032*		−0.16	−0.39 to 0.07	0.168	
	2010	−0.20	−0.43 to 0.03	0.092		−0.14	−0.38 to 0.10	0.261	
	2011	−0.33	−0.57 to −0.09	*0.007*		−0.21	−0.46 to 0.04	0.106	
	2012	−0.07	−0.34 to 0.20	0.596		0.01	−0.26 to 0.29	0.974	
	2013	−0.41	−0.80 to −0.02	*0.040*		−0.07	−0.49 to 0.35	0.734	
*Stunting*									
Prevalence^a^		−0.03	−0.08 to 0.02	0.254	0.254	−0.03	−0.09 to 0.03	0.343	0.343
HAZ SD^a^		−0.06	−0.12 to 0.01	0.076	0.076	−0.08	−0.14 to −0.01	*0.023*	*0.023*
Mean cluster size		0.03	0.02 to 0.04	*<0.001*	*<0.001*	0.04	0.02 to 0.06	*<0.001*	*<0.001*
Location	West Africa	–	–	–	*0.043*	–	–	–	*0.001*
	East Africa	−0.00	−0.35 to 0.35	0.995		−0.12	−0.43 to 0.20	0.475	
	Central/Southern Africa	0.02	−0.39 to 0.42	0.942		0.09	−0.27 to 0.45	0.619	
	Democratic Republic of Congo	0.25	−0.06 to 0.56	0.112		−0.25	−0.60 to 0.10	0.158	
	Sudan	0.36	0.03 to 0.70	*0.036*		−0.20	−0.55 to 0.14	0.255	
	Middle East	0.55	0.10 to 1.00	*0.016*		0.53	0.13 to 0.93	*0.010*	
	South Asia	0.09	−0.29 to 0.48	0.629		0.34	−0.03 to 0.72	0.076	
	Americas	0.17	−0.32 to 0.66	0.493		0.33	−0.12 to 0.78	0.152	
Survey year	2006	–	–	–	*0.010*	–	–	–	0.068
	2007	−0.36	−0.64 to −0.09	*0.011*		−0.18	−0.43 to 0.07	0.160	
	2008	−0.08	−0.33 to 0.17	0.521		0.11	−0.13 to 0.34	0.365	
	2009	−0.41	−0.70 to −0.11	*0.007*		−0.10	−0.39 to 0.20	0.518	
	2010	−0.31	−0.63 to 0.00	0.053		−0.03	−0.33 to 0.28	0.864	
	2011	−0.47	−0.80 to −0.13	*0.006*		−0.32	−0.65 to 0.01	0.062	
	2012	−0.17	−0.54 to 0.19	0.351		0.07	−0.27 to 0.41	0.680	
	2013	−0.63	−1.16 to −0.10	*0.021*		−0.36	−0.89 to 0.16	0.179	

Values in italics represent statistical significance at the 0.05 level

^a^Coefficients and confidence intervals for prevalence and SD are scaled to represent a 0.1 unit change in prevalence and SD

#### Wasting

Univariable analyses for wasting revealed that prevalence, SD of WHZ, mean cluster size, survey location, and survey year were all significantly associated with DEFF. In the multivariable model for wasting, a 0.10 unit increase in prevalence was significantly associated with a 0.27 unit increase in DEFF (95% CI 0.19 to 0.35, p < 0.001). Similarly, an increase in mean cluster size was significantly associated with an increase in DEFF, with every one person increase in mean cluster size being associated with an increase of 0.02 in DEFF (95% CI 0.00 to 0.03, p = 0.013). Location was significantly associated with DEFF (p < 0.001) as seen in Table 6, and certain locations including the Middle East and South Asia were significantly higher when compared to DEFFs in West Africa. Although not significant as a whole (p = 0.102), survey year was significantly related to decreased DEFFs for the years 2010 (β = −0.23, 95% CI −0.43 to −0.03) and 2011 (β = −0.34, 95% CI −0.49 to −0.08) when compared with 2006. Increasing SD of the WHZ distribution was significantly related to increasing DEFF: for every 0.10 unit increase in SD, DEFF increased by approximately 0.10 units (95% CI 0.03 to 0.17, p = 0.009). The overall fit of the multivariable model for wasting, assessed via the adjusted R^2^ value, was 0.24.

#### Underweight

Univariable analyses for underweight show that prevalence, SD of WAZ, mean cluster size, survey location, and survey year were all significantly associated with DEFF. As for wasting, in the multivariable model for underweight increased mean cluster size and increased prevalence were both significantly associated with an increase in DEFF (p < 0.001 for both). Location was significantly associated with DEFF for underweight (p = 0.004); both the Americas and the Middle East were significantly associated with increased DEFFs when compared to West Africa (p = 0.010 and p = 0.002, respectively). Similar to the model for wasting, survey year in the underweight model was as a whole not significantly associated with DEFF (p = 0.086), although surveys conducted during 2007 had significantly lower DEFFs when compared to 2006 (p = 0.045). SD of WAZs was positively associated with DEFF. However, this relationship was only significant in the univariable model, a contrast to the relationship in the model for wasting. The overall fit of the multivariable model for underweight, assessed via the adjusted R^2^ value, was 0.18.

#### Stunting

In the univariable models for stunting, only survey year, survey location and mean cluster size were significantly associated with DEFF. In the multivariable model, as for both wasting and underweight, increased mean cluster size was significantly associated with an increase in DEFF (p < 0.001). Similarly, location was significantly associated with DEFF for stunting (p = 0.001); specifically, the Middle East was significantly associated with increased DEFFs when compared to West Africa (p = 0.010). Similar to the models for both wasting and underweight, survey year in the stunting model was as a whole not significantly associated with DEFF (p = 0.068). In contrast to what was seen in both the wasting and underweight models, prevalence was not significantly associated with stunting DEFFs. Finally, continuing the inconsistent trend in the relationship between DEFF and SD, a 0.1 unit increase in SD of HAZ was associated with a significant 0.08 unit decrease in DEFF for stunting (95% CI −0.14 to −0.01, p = 0.023); notably, this relationship was non-significant in the univariable model. The overall fit of the multivariable model for stunting, assessed via the adjusted R^2^ value, was 0.15.

## Discussion

This is the first review of DEFF for child anthropometric indicators across small-scale nutrition surveys in emergency settings since the release of the new SMART guidelines and WHO Growth Standards in 2006. Consistent with current field survey guidance recommending the use of a DEFF of 1.5 for wasting in the absence of information on prevalence and DEFFs from previous surveys, evidence presented here suggests that median DEFF for wasting was approximately 1.35 [19, 31, 32]. DEFF for wasting fell below 1.5 the majority of the time, suggesting that in most settings estimating sample size based on this value would allow for a sufficiently large sample to achieve desired precision. This finding supports previous research findings that DEFFs for nutrition indicators routinely fall below 2.0 [8, 9]. Where underweight or stunting are the primary indicator of interest, as may be the case in more stable settings, a higher DEFF should be expected. The proportion of surveys with DEFF less than 1.5 for wasting (63%) is approximately the same as the proportion of surveys for stunting (62%) and underweight (71%) with a DEFF less than 2.0. This relationship was consistent across all regions and years, providing further evidence to consider a larger DEFF when underweight or stunting rather than wasting are the primary outcomes of interest. Our evidence suggests that a DEFF of 2.0 may be an appropriate estimate to use in sample size calculations in the absence of other information for these two indicators.

Prevalence of wasting observed in the surveys included in this analysis ranged from 0% to values well exceeding emergency thresholds (max: 38%) [33]. As expected, the median prevalence of wasting (10%) was lower than that for underweight (27%) or stunting (42%) [34]. The prevalences of underweight and stunting were closer to 50% than for wasting, which may in part explain the higher values of DEFF for underweight and stunting observed [2].

The SD of WHZ and WAZ were approximately 1.00, as expected in high-quality anthropometry surveys (WHZ median = 1.03, WAZ median = 1.04). The SDs for HAZ were on average higher than those for WHZ or WAZ. As noted, SD of Z-scores is considered a measure of both heterogeneity as well as anthropometric data quality. It has been observed that SD for HAZ is often greater than WAZ given the greater difficulty of measuring height relative to weight since the introduction of electronic scales. In addition, in contexts where date of birth is unknown and age is therefore estimated, the imprecisions in age determination add additional random variability to the data and SD for HAZ may be expected to be wider than for WHZ [31].

As a parameter used to calculate DEFF, mean cluster size was included in our statistical models. We observed a gradual, but significant decline in mean cluster size over the period studied. This decline is likely a response to the 2006 release and gradual implementation of the SMART guidelines for small-scale field emergency nutrition surveys which recommended individualized sample size calculations for each survey rather than a prescribed standard cluster size of 30 children [11, 32]. This trend occurred in parallel with a significant increase in the mean number of clusters. The shift to a larger number of smaller clusters in more recent years has resulted in an overall decrease in sample size.

The models presented here for DEFF confirm empirically what can be illustrated mathematically from the DEFF formula—that mean cluster size is positively associated with DEFF. Mean cluster size was significantly positively related to DEFF for all three anthropometry indicators. This is important to consider when designing a survey, as the impact of a change in mean cluster size can be sizable depending on the magnitude of the change. Our modeling suggests that reducing the mean cluster size from the formerly prescribed 30 children to 20 children would decrease the DEFF by 0.20–0.40 on average, depending on the indicator.

As expected, prevalence was also significantly associated with DEFFs for wasting and underweight. An increase in DEFF related to a 0.1 increase in prevalence is quite large—on the scale of 0.1–0.3, depending on indicator. This is essential to consider in the survey design phase as regions with an anticipated high prevalence of wasting or underweight, such as in some acute emergency settings, may exhibit higher DEFFs, thereby requiring higher sample sizes. Previous research has demonstrated that the increase in DEFF is more gradual as prevalence nears 50% compared to the change at lower prevalences [2]. Given that our median stunting prevalence was 42%, this may have contributed to the lack of significance in the association between DEFF and prevalence for stunting, a contrast to the relationship observed for wasting and underweight for which median prevalences were lower [34].

A significant positive relationship between DEFF and SD of the Z-scores was observed in the model for wasting, an interesting phenomenon not previously described. A 0.1 unit increase in the SD of WHZ would result in an increase of approximately 0.1 in DEFF. However, the model for stunting suggests a significant relationship of similar strength in the reverse direction, such that a 0.1 unit increase in SD of HAZ would result in a 0.08 unit decrease in DEFF. It is unclear why the directionality of the relationship between SD and DEFF was opposite in these two models, and requires further research to fully understand. However, despite the preliminary nature of these findings, these have important implications on survey design, particularly for wasting which is frequently the outcome of interest in anthropometric surveys. In situations where data quality is anticipated to be low, it is recommended that DEFFs be estimated more conservatively in order to take into account the loss of statistical efficiency due to increased WHZ SDs, and therefore increased DEFFs.

Location and year were also significantly associated with DEFF. While these are generally not modifiable parameters, this highlights the importance of researching the results of previous studies in the same area prior to calculating sample size. The finding that surveys conducted in the Middle East were associated with significantly higher DEFFs for all three indicators further reinforces this. Survey year was significantly associated with DEFF for stunting, and certain years were significant in the other two models. This may in part be a factor of the variability in the number of surveys per location per year, and thus an interaction term in the multivariable models may have better captured this relationship. However, in order to maintain interpretability of the models, no interaction terms were included.

There are a number of limitations to our analyses. First, the adjusted R^2^ value for each of the three models was quite low, indicating that a large part of the variability in DEFFs was not explained by the models, especially for stunting. Second, this analysis only includes surveys conducted by ACF; including field surveys conducted by other agencies would make this analysis more comprehensive and generalizable. Finally, most countries were grouped broadly into regions based on the number of surveys and their general geographic location, but changes in these groupings may alter the results, particularly as the number of surveys was not equal across all regions. However, when the models were run using individual countries rather than geographical grouping of regions, these results did not change substantially (data not shown).

## Conclusions

This research provides evidence as to the magnitude and variation in DEFF observed in small-scale nutrition surveys. Our analyses suggest that for anthropometric surveys focused on wasting, estimating that the expected DEFF will be approximately 1.50 is appropriate in the absence of more context specific information. For stunting and underweight, a higher estimate should be considered. However, given the observed relationship between region and DEFF, this study highlights the need to adapt the global guidance to each context and ideally take into consideration region- or country-specific estimates observed in previous surveys.

The DEFF models provide empirical evidence of a positive relationship between DEFF and both mean cluster size and prevalence. They further provide new evidence of factors related to DEFF, the most notable of which is the demonstration of a significant relationship between SD of the underlying continuous variable and DEFF of the derived categorical variable, even after controlling for other predictors. Further research is needed to better understand why the directionality of this relationship is not consistent across all outcomes.

While these models are not intended to be used for prediction given the relatively low adjusted R^2^ values, they provide important insights into the magnitude and directionality of the effect of each of the predictor variables. As such, these results can inform the survey design decisions of what value of expected DEFF to use in estimating sample size; survey designers should utilize DEFFs from surveys conducted recently in similar regions as a starting point, but should also consider the magnitude of effect observed for each of the predictors in the models to adjust these DEFFs accordingly.

## Abbreviations

ACC/SCN: United Nations Administrative Committee on Coordination/Sub-Committee on Nutrition
ACF: Action Contre la Faim
CDC: US Centers for Disease Control and Prevention
DEFF: design effect
DHS: Demographics and Health Survey
HAZ: height-for-age Z-scores
ICC: intracluster correlation coefficient
MICS: Multiple Indicator Cluster Survey
SMART: Standardized Monitoring and Assessment of Relief and Transitions
SRS: simple or systematic random sample
UNICEF: United Nations Children’s Fund
USAID: United States Agency for International Development
VIF: variance inflation factor
WAZ: weight-for-age Z-scores
WHO: World Health Organization
WHZ: weight-for-height Z-scores

## References

[1] Bilukha O, Blanton C (2008). Interpreting results of cluster surveys in emergency settings: is the LQAS test the best option?. Emerg Themes Epidemiol.

[2] Katz J, Zeger SL (1994). Esimtation of design effects in cluster surveys. Ann Epidemiol.

[3] United Nations Children’s Fund (UNICEF). Multiple Indicator Cluster Survey (MICS). http://mics.unicef.org/

[4] United States Agency for International Development (USAID). Demographic and Health Surveys (DHS). The DHS Program.

[5] Bilukha O (2008). Old and new cluster designs in emergency field surveys: in search of a one-fits-all solution. Emerg Themes Epidemiol..

[6] Kish L (1965). Survey sampling.

[7] Ukoumunne OC, Gulliford MC, Chinn S, Sterne JA, Burney PG (1998). Methods for evaluation area-wide and organization-based interventions in health and health care: a systematic review. Health Technol Assess.

[8] Deitchler M, Deconinck H, Bergeron G (2008). Precision, time, and cost: a comparison of three sampling designs in an emergency setting. Emerg Themes Epidemiol.

[9] Kaiser R, Woodruff BA, Bilukha O, Spiegel PB, Salama P (2006). Using design effects from previous cluster surveys to guide sample size calculation in emergency settings. Disasters.

[10] United Nations Administrative Committee on Coordination/Sub-Committee on Nutrition (ACC/SCN). Report of a workshop on the improvement of the nutrition of refugees and displaced people in Africa. ACC/SCN, Machakos. 1995.

[11] Standardized Monitoring and Assessment of Relief and Transitions (SMART). Measuring mortality, nutritional status, and food security in crisis situations, Version 1. 2006.

[12] Bennett S, Woods T, Liyanage WM, Smith DL (1991). A Simplified general method for cluster-sample surveys of health in developing countries. World Health Stat Q.

[13] World Health Organization (WHO). Physical status: the use and interpretation of anthropometry. WHO technical report series 854. Geneva: WHO; 1995. http://apps.who.int/iris/handle/10665/37003. 8594834

[14] Mei Z, Grummer-Strawn LM (2007). Standard deviation of anthropometric Z-scores as a data quality assessment tool using the 2006 WHO growth standards: a cross country analysis. Bull World Health Organ.

[15] De Onis M (2006). WHO child growth standards: length/height-for-age, weight-for-age, weight-for-length, weight-for-height and body mass index-for-age: methods and development.

[16] Action Contre la Faim (ACF) International. http://www.actioncontrelafaim.org/.

[17] Action Contre la Faim (ACF) (2015). Anthropometric survey data, 2001–2013.

[18] Grellety E, Golden MH (2016). Weight-for-height and mid-upper-arm circumference should be used independently to diagnose acute malnutrition: policy implications. BMC Nutr.

[19] Standardized Monitoring and Assessment of Relief and Transitions (SMART). Sampling methods and sample size calculation for the SMART methodology. 2012. http://smartmethodology.org/survey-planning-tools/smart-methodology/.

[20] SAS Institute (2014). SAS software version 9.3 for windows.

[21] Salganik MJ (2006). Variance estimation, design effects, and sample size calculations for respondent-driven sampling. J Urban Health Bull N Y Acad Med.

[22] Team R (2015). RStudio: integrated development for R.

[23] Neter J, Kutner M, Nachtsheim C, Wasserman W (1996). Applied linear regression models.

[24] Faraway J (2004). Chapter 4—diagnostics. Linear models with R.

[25] Wickham H (2009). ggplot2: elegant graphics for data analysis.

[26] Wickham H (2011). The split-apply-combine strategy for data analysis. J Stat Softw.

[27] Wickham H (2007). Reshaping data with the reshape package. J Stat Softw.

[28] Standardized Monitoring and Assessment of Relief and Transitions (SMART). The SMART plausibility check for anthropometry. 2015. http://smartmethodology.org/survey-planning-tools/smart-methodology/.

[29] Neter J, Wasserman W, Kutner MH (1989). Applied linear regression models.

[30] Rogerson PA (2001). Statistical methods for geography.

[31] United Nations High Commissioner for Refugees (UNHCR). Standardised expanded nutrition survey (SENS) guidelines for refugee populations, version 2. 2013. http://sens.unhcr.org/wp-content/uploads/2015/03/UNHCR_SENS_Pre-Module_v2.pdf.

[32] United Nations Children’s Fund (UNICEF). Division of policy and planning: multiple indicator cluster survey manual 2005—monitoring the situation of children and women. New York; 2005. http://mics.unicef.org/tools?round=mics3.

[33] United Nations High Commissioner for Refugees (UNHCR). Acute malnutrition threshold. In: UNHCR emergency handbook. 4th ed. 2015. https://emergency.unhcr.org/entry/32605/acute-malnutrition-threshold.

[34] Crowe S, Seal A, Grijalva-Eternod C, Kerac M (2014). Effect of nutrition survey ‘cleaning criteria’ on estimates of malnutrition prevalence and disease burden: secondary data analysis. PeerJ.

